# Colorant Pigments, Nutrients, Bioactive Components, and Antiradical Potential of Danta Leaves (*Amaranthus lividus*)

**DOI:** 10.3390/antiox11061206

**Published:** 2022-06-20

**Authors:** Umakanta Sarker, Md. Asif Iqbal, Md. Nazmul Hossain, Shinya Oba, Sezai Ercisli, Crina Carmen Muresan, Romina Alina Marc

**Affiliations:** 1 Department of Genetics and Plant Breeding, Faculty of Agriculture, Bangabandhu Sheikh Mujibur Rahman Agricultural University, Gazipur 1706, Bangladesh; asifiqbal2238@gmail.com (M.A.I.); nnazmul99@gmail.com (M.N.H.); 2 Laboratory of Field Science, Faculty of Applied Biological Sciences, Gifu University, Yanagido 1-1, Gifu 501-1193, Japan; soba@gifu-u.ac.jp; 3 Department of Horticulture, Faculty of Agriculture, Ataturk University, 25240 Erzurum, Turkey; sercisli@atauni.edu.tr; 4 Food Engineering Department, Faculty of Food Science and Technology, University of Agricultural Sciences and Veterinary Medicine, 400372 Cluj-Napoca, Romania; crina.muresan@usamvcluj.ro

**Keywords:** danta, foliage by-products, bioactive components, nutrients, colorant pigments, polyphenols, antioxidants

## Abstract

In the Indian subcontinent, danta (stems) of underutilized amaranth are used as vegetables in different culinary dishes. At the edible stage of the danta, leaves are discarded as waste in the dustbin because they are overaged. For the first time, we assessed the colorant pigments, bioactive components, nutrients, and antiradical potential (AP) of the leaves of danta to valorize the by-product (leaf) for antioxidant, nutritional, and pharmacological uses. Leaves of danta were analyzed for proximate and element compositions, colorant pigments, bioactive constituents, AP (DPPH), and AP (ABTS^+^). Danta leaves had satisfactory moisture, protein, carbohydrates, and dietary fiber. The chosen danta leaves contained satisfactory magnesium, iron, calcium, potassium, manganese, copper, and zinc; adequate bioactive pigments, such as betacyanins, carotenoids, betalains, β-carotene, chlorophylls, and betaxanthins; and copious bioactive ascorbic acid, polyphenols, flavonoids, and AP. The correlation coefficient indicated that bioactive phytochemicals and colorant pigments of the selected danta leaves had good AP as assessed via ABTS^+^ and DPPH assays. The selected danta leaves had good ROS-scavenging potential that could indicate massive possibilities for promoting the health of the nutraceutical- and antioxidant-deficit public. The findings showed that danta leaves are a beautiful by-product for contributing as an alternate origin of antioxidants, nutrients, and bioactive compounds with pharmacological use.

## 1. Introduction

Amaranth is a promising crop with a wide divergence [1,2,3,4,5,6,7] Amaranth contains 20 times more calcium, 13 times more extra ascorbic acid, 7 times more iron, and 18 times more vitamin A precursor than lettuce [8]. This rapidly growing crop has C_4_ photosynthesis and versatile uses, namely as vegetables, ornamentals, and grains. It has broad adaptation and is dispersed in the United States of America, Africa, Europe, Asia, and Australia. Amaranth is a low-priced vegetable. Its stems and leaves are edible and have copious ascorbic acid; protein, with lysine and methionine amino acids essential for human nutrition [9,10,11,12,13,14,15,16,17]; carotenoids; digestible fiber; and minerals, including calcium, copper, magnesium, zinc, potassium, iron, and manganese [18,19,20,21,22,23,24,25,26,27,28,29,30,31,32,33,34,35,36,37,38,39,40,41,42,43,44,45]. *Amaranthus* has been used as folk medicine, particularly in anthelminthic [46,47,48], antimicrobial [49,50,51,52,53,54,55,56,57,58], anti-inflammatory [59,60,61], anticancer [62,63,64,65], hepatoprotective [66,67,68,69], antilipidemic [70,71,72,73,74,75], antimalarial [76], antidiabetic [77], antiviral [78], neuroprotective [79], antiulcer [80], and snake antidote contexts, among others. [81,82,83,84,85]. It also has abundant pigments, including carotenoids, betacyanins, betalains, betaxanthins, and chlorophylls [86,87,88,89,90,91,92,93,94,95,96,97] with high AP [98,99,100,101,102,103,104,105,106,107,108,109,110,111], phytochemicals including ascorbic acids, flavonoids, phenolic acids [112,113,114,115,116,117] with high AP [118,119,120,121,122,123,124,125,126,127,128,129,130]. These natural-origin compounds can quench reactive oxygen species (ROS) [131,132,133,134,135,136,137,138,139,140,141,142,143,144,145,146,147,148,149] and predominantly influence the industry of foods [96,97]. Pigments of amaranth are able to efficiently quench radicals [103,104,105,106,107,108,109]. Amaranth is widely acclimated to the environmental stresses of drought [150,151,152,153,154,155,156,157,158] and salinity [159,160,161,162,163].

The scarcity of calories and food insecurity has resulted in malnourishment in 79.5 crore people worldwide [164]. Approximately two crore people are affected by hidden hunger due to deficiency of minerals or vitamins [165]. Staple foods are the principal source of energy but have deficits of iron, α-carotene, zinc, β-carotene, iodine, other carotenoids, ascorbic acid, and vitamin E [166]. Consuming staple foods regularly results in hidden hunger [165]. However, consuming staple foods alongside vegetables and fruits ensures a healthy diet with a balanced vitamin and mineral source. Phytochemical compounds, including ascorbic acid, pigments, and phenolics, have significant contributions to health promotion [167,168,169].

Currently, antioxidants from natural sources, especially vegetables, have attracted the attention of consumers and researchers. Amaranth bioactives comprise β-carotene, flavonoids, vitamin C, and phenolics, which have radical quenching potential [96,97]. These bioactive components defend against numerous diseases, such as cataracts, cardiovascular diseases, atherosclerosis, emphysema, arthritis, cancer, retinopathy, and degenerative diseases of the neuron [169,170,171,172]. Natural products capable of antioxidant properties have gained substantial interest.

Danta (*Amaranthus lividus*) is a wild and edible vegetable. It is found as wild vegetables throughout the world, including in its native habitat. However, in the Indian subcontinents, it has grown as a wild and cultivated form. The diversified germplasm of amaranth indicates its enormous variability and plasticity in phenotype [173,174,175,176,177,178,179,180,181,182,183,184,185,186,187,188,189,190,191,192,193,194,195], which has multipurpose uses. The attractive flavor, color, and taste make it a standard vegetable in different culinary dishes in the Indian subcontinent. It can be grown throughout the year on the Indian subcontinent in vegetable gap periods between winter and hot summer [9,10]. The succulent, juicy, and sizeable barreled stem becomes edible as a vegetable 50–60 days after sowing and can be consumed until seed maturity. This stem is popularly known as danta. Generally, flowering starts at around 2–3 months of age, though cultivars sensitive to the photoperiod flower at around 9 months to 1 year. The big and flashy stem is consumed year-round as a very famous vegetable in India and Bangladesh. The leaves of danta are not consumed, because at the time of harvesting of danta (the edible stage of the stem), the leaves are aged. They are thus left in the dustbin as waste materials. However, the by-product leaves have an attractive ROS scavenging potential. They could be used as an alternate origin of nutrients and bioactive compounds for the benefits of health promotion and nourishing the people’s scarcity in antioxidants and nutraceuticals. The literature has shown that amaranth leaves are a plentiful origin of pigments, nutrients, antioxidants, minerals, and phytochemicals compared with the stem [96,108]. For this reason, we herein investigated the possibility of exploiting danta leaves as a source of nutraceuticals and natural pigments because of their sufficient betacyanins, betaxanthins, betalains, and phytochemicals of interest in the food industry [93,94,95]. Therefore, for the first time, we investigated the nutraceuticals, phytochemicals, pigments, and antioxidant potential of the selected danta leaves to valorize the leaves as antioxidants, bioactive compounds, and nutrient sources.

## 2. Materials and Methods

### 2.1. Materials of the Study

The Department of Genetics and Plant Breeding of Bangabandhu Sheikh Mujibur Rahman Agricultural University preserved many amaranth germplasm accessions in the gene bank. In our previous studies, we evaluated many amaranth accessions for their agronomic and antioxidant potentials [9,10,11,12,95]. Finally, four advanced accessions (SA5, SA8, SA9, and SA17) were selected based on the yields and antioxidant potentials evaluated in our previous studies.

### 2.2. Layout and Design

The investigation was carried out following a randomized design with three blocks (3 replicates) at BSMRAU. Each experimental unit comprised a 1 m^2^ plot using row and plant spacing of 25 cm and 10 cm, respectively.

### 2.3. Intercultural Practices

Appropriate cultural practices and recommended doses of fertilization with inorganic fertilizer and organic compost were maintained. The spacing of plants was continued following appropriate thinning. Weeds were eradicated with hoeing at regular intervals. Regular irrigation was provided to uphold the adequate growth of plants. At 60 days of age, the leaf samples were collected from plants. The leaves were washed thoroughly in tap water. Water on the surface of washed leaves was removed by spreading the leaves on a clean board in a well-ventilated room at room temperature until the water was removed from the leaves through evaporation. Then, leaves were used for further phytochemical extraction from the fresh sample.

### 2.4. Reagents and Solvents

Reagents: H_2_SO_4_, cesium chloride, dithiothreitol (DTT), HClO_4_, ascorbic acid, HNO_3_, ABTS^+^, Trolox, Folin–Ciocâlteu reagent, rutin, DPPH, 2,2-dipyridyl, AlCl_3_6H_2_O, gallic acid, CH_3_CO_2_K, potassium persulfate, and Na₂CO₃. Solvent: Methanol, hexane, and acetone. All solvents and reagents used in this study were high purity laboratory products obtained from Kanto Chemical Co. Inc. (Tokyo, Japan) and Merck (Darmstadt, Germany).

### 2.5. Estimation of Proximate Composition

Ash, fat, moisture, fiber, protein, and energy were determined using the AOAC method [196]. Nitrogen was calculated following the micro-Kjeldahl method (AOAC method 976.05). The protein was determined by multiplying the N value by 6.25. The total fat, ash, moisture, and protein (%) were deducted from one hundred (100) to estimate the carbohydrates (g 100 g^−1^ fresh weight (FW)).

### 2.6. Estimation of Mineral Composition

The leaves were dried for 24 h in an oven maintaining 70 °C temperature and ground in a mill. Magnesium, potassium, calcium, manganese, copper, zinc, and iron were determined from leaf powder. The samples (0.5 g) were digested with 400 mL HNO_3_ (65%), 40 mL HClO_4_ (70%), and 10 mL H_2_SO_4_ (96%) [197]. An atomic absorption spectrophotometry (AAS) device (Hitachi, Tokyo, Japan) was used to read the absorbance [61] at 285.2 (Mg), 213.9 (Zn), 766.5 (K), 248.3 (Fe), 279.5 (Mn), 422.7 (Ca), and 324.8 (Cu) nm wavelengths.

### 2.7. Carotenoids and Chlorophylls Determination

Chlorophylls (*ab*, *b*, and *a*) and carotenoids were determined by extracting the samples in C_3_H_6_O (80%) [198]. A Hitachi spectrophotometer (Tokyo, Japan) was used to estimate the absorbance at 646, 663, and 470 nm for carotenoids and chlorophyll *b* and *a*, respectively. Carotenoids were calculated as milligrams per 100 g, and chlorophyll was calculated as micrograms per gram of FW.

### 2.8. Determination of Betacyanins and Betaxanthins

We extracted the leaves in MeOH (80%) comprising ascorbic acid (50 mM) [199,200,201]. The betacyanins and betaxanthins were estimated using a spectrophotometer at 540 and 475 nm wavelengths. The results were calculated as nanograms of betanin and indicaxanthin equivalent per gram of FW for betacyanins and betaxanthins.

### 2.9. Estimation of β-Carotene

Exactly 500 mg of fresh leaves was thoroughly ground with 10 mL C_3_H_6_O (80%) in a mortar and pestle and centrifuged for 3–4 min at 10,000 × *g* to estimate β-carotene [150]. The supernatant was transferred to a volumetric flask and marked up to 20 mL. A Hitachi spectrophotometer (Tokyo, Japan) was used to take the absorbance at 510 and 480 nm, respectively. β-carotene was determined as mg of β-carotene/100 g FW.

### 2.10. Estimation of Ascorbic Acid

A spectrophotometer was set to determine DHA and AsA from fresh samples of the leaf. Dithiothreitol (DTT) was utilized to preincubate the sample. Dithiothreitol (DTT) reduced dehydroascorbic acid to ascorbic acid. As a result of the ascorbic acid reduction, a ferrous ion was formed from the ferric ion. Reduced ferrous ions react with 2,2-dipyridyl to form complexes [150]. To estimate ascorbic acid, the absorbance of Fe^2+^ complexes with 2,2-dipyridyl was read at 525 nm using a spectrophotometric (Hitachi, Tokyo, Japan). The ascorbic acid was calculated in mg/100 g FW.

### 2.11. Extraction of Samples and Estimation of TP, AP, and TF

The fresh and dried ground leaves (60 d) were used to produce extract in a mortar and pestle for estimation of AP, total flavonoids (TF), and total polyphenols (TP). Leaves (0.25 g) were added in 90% MeOH (10 mL) in a tightly capped bottle and placed at 60 °C in a water bath (Tokyo, Japan) for 1 h. We filtered the extract and kept it for the estimation of AP, TF, and TP. Folin–Ciocâlteu reagent and the aluminum chloride colorimetric method were used to estimate polyphenols [202] and flavonoids [203,204], respectively. A spectrophotometer (Hitachi, Tokyo, Japan) was used to take the absorbance at 760 and 415 nm for TP and TF, respectively. A standard gallic acid curve (Y = 0.009X + 0.019) and rutin curve (Y = 0.013X) were made, and TP and TF were estimated as μg GAE g^−1^ of FW and μg RE g^−1^ DW, respectively. The diphenyl-picrylhydrazyl (DPPH) radical degradation method was used to estimate AP (DPPH) [150]. The method of Khanam et al. [205] was used to perform the ABTS^+^ assay. DPPH and ABTS^+^ inhibition percentages corresponding to the control were followed to estimate AP using the equation:

AP (%) = (AB − ALS/AB) × 100

where AB is the optical density of the blank [as a substitute of leaf extract 10 µL and 150 μL MeOH for AP (DPPH and ABTS), respectively] and ALS is the optical density of the leaf samples. The data are expressed as Trolox equivalent (TE) μg g^−1^ DW.

### 2.12. Statistical Analysis

The averaged data from each replication constituted the replication mean. The Statistix 8 software was used to analyze the data for analysis of variance (ANOVA) [206,207,208]. The means data were compared using the Duncan multiple range test at a probability of 1%. The data are presented as the mean ± SD.

## 3. Results and Discussion

Analysis of variance indicated noteworthy variation among parameters for all characters. Widespread differences were also revealed in the biochemical traits of amaranth [197,198,199].

### 3.1. Composition of Proximate

**Figure 1:**
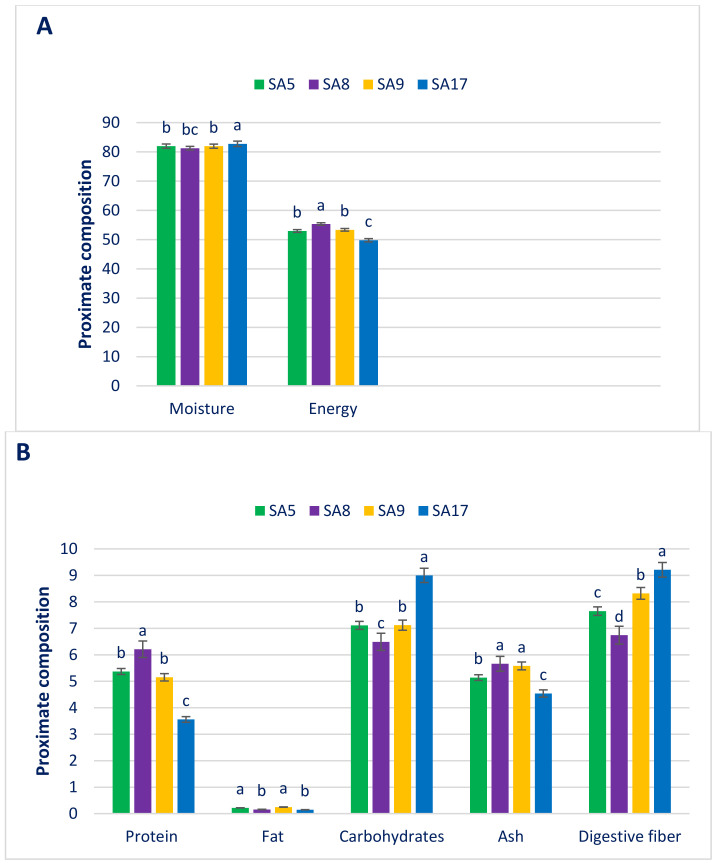
Proximate composition (per 100 g^−1^ FW, energy kcal 100 g^−1^ FW) in danta leaves. (**A**) Moisture and energy, (**B**) Protein, fat, carbohydrates, ash and digestive fiber. Dissimilar letters over the bars indicate that the corresponding data significantly differed by Duncan multiple range test (DMRT) (*p* < 0.01, n = 6).

Figure 1 shows the composition of moisture, fat, carbohydrates, protein, ash, fiber (g 100 g^−1^ FW), and energy (kcal 100 g^−1^ FW) of danta leaves. The moisture of danta leaves differed from 81.47 in SA8 to 82.75 in SA17. As lower moisture content confers higher leaf dry matter; hence, some accessions had considerable dry biomass (18–19% dry mass). The moisture of leaves is straightly interrelated to maturity. The results from these danta leaves were in agreement with those from *A. tricolor* [10] and sweet potato leaves [209].

As for vegetables, danta leaves had a high protein that prominently differed among accessions (3.56 to 6.21). As leafy vegetables, greater protein content was observed in SA8, SA5, and SA9. Vegetarians and poor people in underdeveloped countries mostly trust danta leaves as a source of protein. Danta leaves displayed a much greater protein content than *A. tricolor* (1.26%) [10]. Danta leaves displayed low fat content owing to being a vegetable and can be consumed as a fat-free food. Danta genotypes varied significantly for leaf fat content (0.15–0.25), which results were corroborated by those for *A. tricolor* [10] and sweet potato [209]. The authors of [10,209] noted that fat upholds the temperature of the body, covers organs, and influences cell function. Fats are sufficient sources of Ω-6 and Ω-3 fatty acids and give a noteworthy contribution to the transport, digestion, and absorption of the lipid-soluble vitamins K, E, D, and A.

Danta leaves had good carbohydrate content, with ample variation regarding accessions (6.49 to 9.00). SA8 displayed the highest carbohydrate content (9.00), and high carbohydrate content was recorded in SA5 and SA9, while SA17 exhibited the minimum carbohydrates (6.49). The leaves of danta genotypes also had diverse energy content (49.78 to 55.35). Danta leaves of SA8 had the maximum energy (55.35), and high energy was recorded in SA5 and SA9. On the other hand, danta leaves of SA17 had the minimum energy (49.78). SA8 had the highest ash content (5.66); conversely, SA17 had the minimum ash content (4.54).

The dietary fiber also differed among the danta leaves (6.74 to 9.21). Danta leaves of SA17 had the maximum dietary fiber (9.21), followed by SA9. Conversely, SA8 had the minimum dietary fiber (6.74). Dietary fiber remarkably augments digestibility, constipation, and palatability [12]. Danta leaves were rich in protein, dietary fiber, carbohydrates, and moisture. Our earlier study was in agreement with the current findings [10]. The carbohydrate content of the advanced line of danta leaves of SA17 and the protein content of the danta leaves of SA5, SA8, and SA9 were superior to the protein and carbohydrate contents of red amaranth [210], green amaranth [211], weedy amaranth [212], and danta [213]. The dry matter obtained from the advanced line of danta leaves was superior to the dry matter of red amaranth [210], green amaranth [211], *A. spinosus* [212], and danta [213], whereas it was surpassed by the dry matter of *A. viridis* weedy amaranth [212]. The digestible fiber content of the danta leaves of SA17 was higher than that of red, green, and danta [210,211,212,213] but lower than that of weedy amaranth (*A. spinosus*) and comparable to that of weedy amaranth (*A. viridis*) [212].

### 3.2. Mineral Elements

**Figure 2:**
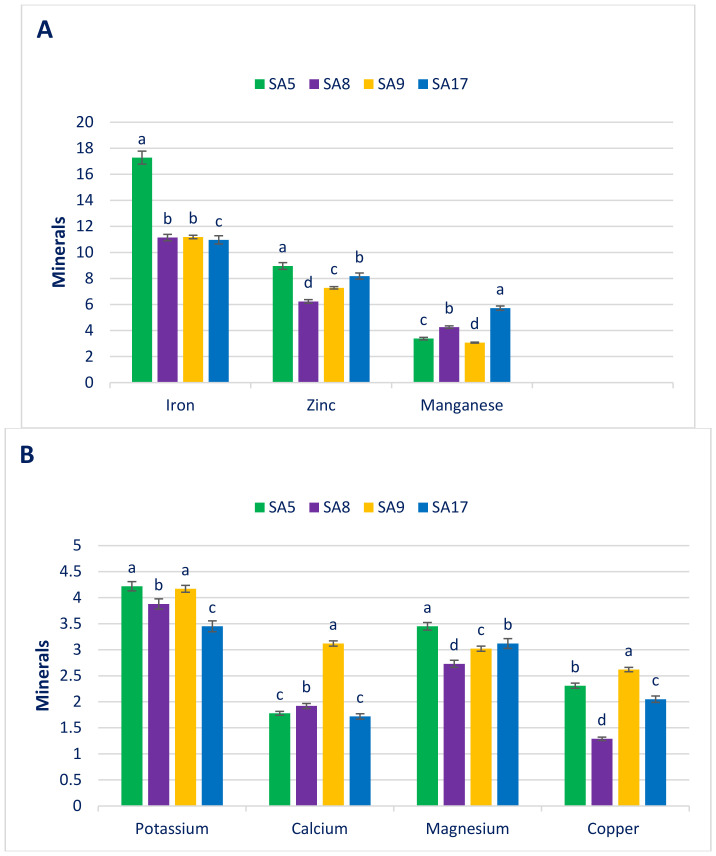
Mineral elements (macroelements mg g^−1^ FW, microelements µg g^−1^ FW) in danta leaves. (**A**) Iron, zinc and manganese, (**B**) Potassium, calcium, magnesium, and copper. Dissimilar letters over the bars indicate that the corresponding data significantly differed by DMRT (*p* < 0.01, n = 6).

Figure 2 shows the mineral elements, both macro- (mg g^−1^ FW) and microelements (µg g^−1^ FW), of the studied danta leaves. The danta leaves had good content of potassium. Danta leaves of SA5 had the maximum potassium (4.22), which was statistically parallel to that of SA9. The minimum potassium was recorded in SA17 (3.45). The calcium greatly varied among accessions (1.72 to 3.12). Danta leaves of SA9 showed the maximum calcium (3.12 mg g^−1^). In contrast, SA17 displayed the minimum calcium (1.72). The danta leaves had good magnesium content, and variations were not prominent regarding accessions (2.73 to 3.45). SA5 had the highest magnesium (3.45). In contrast, SA8 had the minimum magnesium (2.73). We documented sufficient K (4.22), Mg (3.45), and Ca (3.12) in the danta leaves. Several species of amaranth had ample Mg, Ca, and K [202]. These findings also showed that the calcium, potassium, and magnesium in amaranth were much more noticeable than those in spinach, nightshade, black kale, and spider flower. The potassium content of danta leaves was comparable to that of green amaranth [211] but less than that of weedy amaranth [212]. The Ca detected in danta leaves of SA9 was superior to that in weedy amaranth [212], green morph amaranth [211], and *A. blitum* [214]. The magnesium noticed in the danta leaves was superior that of green amaranth [211] and comparable to that of weedy amaranth [212].

Danta leaves displayed wide variations among accessions for Fe content (10.96 to 17.28). SA5 had the maximum Fe (17.28), and SA17, the minimum (10.96). In our study, great differences were observed in the manganese of danta leaves (3.07 and 5.72). SA17 exhibited the highest manganese (5.72), and SA9, the lowest (3.07). The copper had noteworthy differences among the selected danta leaves (1.29 to 2.62). SA9 had the highest Cu (2.62), and SA8 had the lowest Cu (1.29). Zinc content diverged among accessions (6.23 in SA8 to 8.96 in SA5). The iron and zinc content of the selected danta leaves were superior to those of cassava leaves [215] and beach pea [216]. We noted sufficient Fe (17.28), Mn (5.72), and Zn (8.96) and noteworthy Cu (2.62) in the selected danta leaves. In the literature on several species of amaranth, sufficient Mn, Fe, Zn, and Cu were noted [202]. The authors of [202] also showed that leaves of amaranth species had more noticeable Mn, Zn, Fe, and Cu than kale, spinach, spider flower, and black nightshade. In the current investigation, the iron content of danta leaves of SA5 were much superior to that of *A. spinosus* and green amaranth [211,212], even though the iron content of this line was less than that of *A. viridis* [212]. The manganese content of danta leaves was less than that of green and weedy amaranth [211,212]. The copper content of danta leaves was much superior to that of green amaranth [211] but less than that of weedy amaranth [212]. The zinc content of danta leaves was less than that of weedy and green amaranth [211,212].

### 3.3. Bioactive Pigments

**Figure 3:**
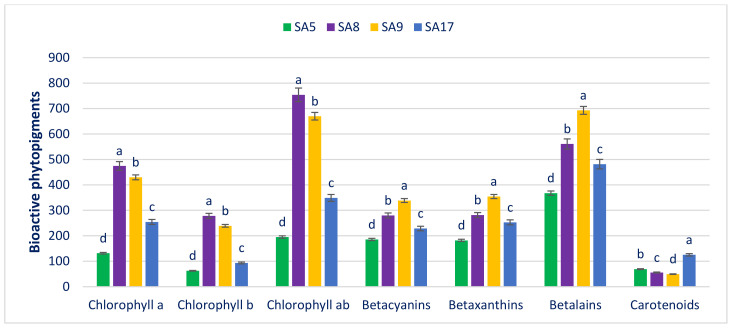
Bioactive pigments in danta leaves. Dissimilar letters over the bars indicate that the corresponding data significantly differed by DMRT (*p* < 0.01, n = 6).

Figure 3 shows the bioactive pigments, such as carotenoids (mg 100 g^−1^ FW), chlorophylls (μg g^−1^ FW), and betalains (ng g^−1^ FW), of danta leaves. Danta leaves exhibited great variation in chlorophyll *a* (ch*a*) content (131.56 to 474.51). SA8 had the highest ch*a* (474.51), followed by SA9. In contrast, SA5 had the lowest ch*a* (131.56). Danta leaves exhibited great variation in chlorophyll *b* (ch*b*) content (62.42 to 278.11). SA8 displayed the highest ch*b* (278.11), followed by SA9. In contrast, SA5 had the lowest ch*b* (62.42). Significant and considerable differences in chlorophyll *ab* (ch*ab*) were observed in danta leaves (194.99 to 753.73). SA8 displayed the highest ch*ab* (753.73), followed by SA9. In contrast, SA5 had the lowest ch*ab* (194.99). Notable contents of ch*a* (474.51), ch*ab* (753.73), and ch*b* (278.11), which was superior to those of green and red amaranth [217], were observed in the selected danta leaves. The observed ch*a*, ch*b*, and ch*ab* levels were much superior to those of red, green, weedy, and danta leaves [210,211,212,213] of our earlier studies.

Danta leaves had good content of betacyanins, with significant variability regarding accessions (185.52 to 338.51). SA9 had the highest betacyanins (338.51). On the other hand, SA5 had the minimum betacyanins (185.52). Danta leaves had good betaxanthins, with noteworthy variability regarding accessions (181.90 to 354.31). SA9 had the highest betaxanthins (354.31). Conversely, SA5 had the minimum betaxanthins (181.90). Danta leaves had good betalain content, with prominent variability regarding accessions (367.35 to 692.74). The betalains were the highest in SA9 (692.74) and the lowest in SA5 (367.35). The carotenoids exhibited significant variation among the accessions (49.39 to 125.17). The highest carotenoids were noted in SA17 (125.17), and the lowest were noted in SA9 (49.39). Notable levels of ch*a* (474.51), carotenoids (125.17), betacyanins (338.51), betalains (692.74), betaxanthins (354.31), and ch*b* (278.11) were recorded in the selected danta leaves, which were comparable to those in green and red amaranth [217]. The betacyanins of danta leaves were much more noticeable than those of green weedy and danta leaves [211,212,213] and comparable to those of red morph amaranth [210]. The betaxanthins and betalains of danta leaves were much more noticeable than those of green morph amaranth [211]. The betaxanthins and betalains in SA9 were much more noticeable than those in red, weedy, and danta leaves [210,211,212,213]. The carotenoid content of SA17 was greater than that of red, green, weedy, and danta leaves [210,211,212,213].

### 3.4. Bioactive Components and AP

**Figure 4:**
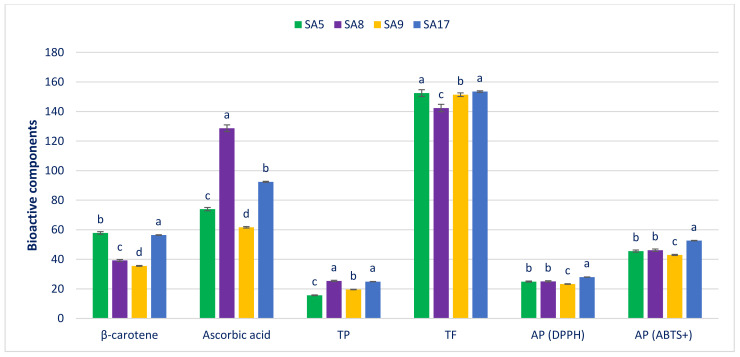
Bioactive components and AP in danta leaves. Dissimilar letters over the bars indicate that the corresponding data significantly differed by DMRT (*p* < 0.01); AP, antiradical potential; TF, total flavonoids; TP, total polyphenols (n = 6).

The total polyphenols (TP, µg g^−1^ FW), β-carotene (mg 100 g^−1^ FW), total flavonoids (TF, μg g^−1^ DW), ascorbic acid (mg 100 g^−1^ FW), and AP (μg g^−1^ DW) of the selected danta leaves are presented in Figure 4. Considerable variation was documented in the β-carotene of the danta leaves (35.54 in SA9 to 57.83 in SA5). The highest β-carotene was observed in SA17 (56.42). The danta leaves also had considerable variation in ascorbic acid (61.63 to 128.68). SA8 displayed the highest ascorbic acid (128.68), and SA9, the lowest (61.63). Noticeable and significant variations were noted in the TP of the selected danta leaves (15.66 to 25.35). SA8 had the highest TP (25.35), followed by SA17; SA5 had the lowest TP (15.66). The selected danta leaves had high TF, with noteworthy differences among genotypes (142.35 to 153.48). SA17 had the highest TF (153.48), which had a statistical similarity to SA5. SA9 had a high TF (151.36), whereas SA8 had the lowest TF (142.35). High DPPH and ABTS^+^ AP were recorded in the danta leaves, with low variability regarding accessions. SA17 had the highest DPPH and ABTS^+^ AP (27.96, 52.64), followed by SA8 (25.06, 46.14) and SA5 (24.92, 45.57).

The minimum ABTS^+^ and DPPH AP were recorded in SA9 (23.26, 42.95). The similar tendencies of AP under the ABTS^+^ and DPPH methods authenticated the antioxidant capacities measured via the two methods. In the current investigation, the selected danta leaves displayed notable ascorbic acid and β-carotene contents (128.68 and 57.83), which were greater than those of red amaranth [10]. The TP (25.35), AP in DPPH (27.96), TF (153.48), and AP in ABTS^+^ (52.64) obtained were substantiated with green and red amaranth [199]. The β-carotene of the danta leaves was comparable to that of weedy amaranth [212] but lower than that previously measured in danta leaves [213] and that in red morph amaranth [210] in our earlier studies. The ascorbic acid obtained from the SA8 danta leaves was superior to that of red, weedy, stem, and green amaranth [210,211,212,213]. The TP of danta leaves was superior to that of green morph amaranth [210] and comparable to that of weedy amaranth (*A. spinosus*) [212]. The TF and AP (ABTS^+^ and DPPH) of the danta leaves were superior to those of green, red, and danta leaves [210,211,214] and comparable to those of weedy amaranth [212]. The selected danta leaves had high levels of antioxidants, phenolics, and flavonoids, along with substantial nutrients, photopigments, and vitamins. These accessions can be used as preferable high-yielding cultivars containing sufficient antioxidants and appropriate for extracting colorful juice. The investigation exposed that danta leaves were a great source of nutrients and phytochemicals with antioxidant activities and presented enormous potential as food for people deficient in minerals, vitamins, and antioxidants.

### 3.5. Association Studies

The relationships of the bioactive pigments, TF, β-carotene, AP (ABTS^+^), TP, AP (DPPH), and ascorbic acid of danta leaves are shown in Table 1. The associations of the bioactive colorant pigments, ascorbic acid, AP (DPPH), TP, β-carotene, TF, and AP (ABTS^+^) of danta leaves had stimulating outcomes. All bioactive colorant pigments were significantly and positively associated with TP, AP (DPPH), TF, and AP (ABTS^+^). This indicated that increases in TP, AP (ABTS^+^), TF, and AP (DPPH) were unswervingly associated with the augmentation of betaxanthins, carotenoids, chlorophylls, betalains, and betacyanins or vice versa. It also confirmed that all bioactive colorant pigments had strong AP.

Likewise, ascorbic acid had significantly positive associations with TP, AP (DPPH), TF, and AP (ABTS^+^), although it displayed negative and insignificant relationships with all bioactive colorant pigments. Sarker and Oba [150,161] detected a parallel tendency. Positive and significant correlations were observed with ascorbic acid, TP, AP (DPPH), TF, β-carotene, and AP (ABTS^+^), which was corroborative to past results on salt-induced amaranth [218,219,220,221,222]. The positive and noteworthy correlations of ascorbic acid, AP (DPPH), TP, β-carotene, TF, and AP (ABTS^+^) suggested that β-carotene, TF, ascorbic acid, and TP had strong AP. The authentication of the AP of the danta leaves by two methods of AP was established, with positive and noteworthy correlations between AP (DPPH) and AP (ABTS^+^). Bioactive pigments and phytochemicals such as β-carotene, TF, TP, and ascorbic acid had strong AP, as confirmed by significant relationships with AP (DPPH) and AP (ABTS^+^). All bioactive pigments, TP, ascorbic acid, TF, and β-carotene displayed vital contributions to the AP of danta leaves, because the compounds had strong AP.

## 4. Conclusions

The leaves of danta leaves had ample sources of Mg, K, carbohydrates, Ca, Fe, dietary fiber, Cu, protein, Zn, and Mn. They were an admirable origin of bioactive pigments such as betacyanins, β-carotene, betalains, ascorbic acid, betaxanthins, carotenoids, TP, chlorophylls, TF, and antioxidants. The correlation coefficient revealed that the bioactive pigments and phytochemicals of danta leaves had good AP (ABTS^+^) and AP (DPPH). Danta leaves are an underutilized but promising vegetable. Danta leaves had enormous bioactive phytochemicals and antioxidants, which could be cultivated in preferable cultivars. The leaves could be utilized as boiled food, fresh salads, leafy vegetables for daily diet, and other culinary dishes. Considering the status of their nutrients, they could be equivalent to spinach. They could also be grown year-round, including in summer, a gap in vegetable growth. The leaves could be used for the extraction of colorful juice as a possible origin of nutritional value, bioactive pigments, phenolics, ascorbic acid, flavonoids, β-carotene, and antioxidants in a regular diet to accomplish antioxidant and nutritional sufficiency. The selected danta leaves contained good ROS-scavenging potential that offered enormous prospects for health promotion in the antioxidant- and nutraceutical-deficit community. We concluded that danta leaves are an attractive by-product to contribute as an alternative source of nutrients, bioactive compounds, and antioxidants.

## Figures and Tables

**Table 1:** The correlation coefficient for ascorbic acid, pigments, TP, β-carotene, AP (DPPH), TF, and AP (ABTS^+^) in danta leaves.

Characters	Ch*b*	Ch*ab*	B	Bn	Bl	βC	AsA	TP	TF	AP	AP
Ch*a*	0.92 **	0.98 **	0.94 **	0.95 **	0.92 **	−0.82 *	−0.024	0.98 **	0.87 *	0.84 *	0.83 *
Ch*b*		0.95 **	0.93 **	0.92 **	0.96 **	−0.71	−0.023	0.81 *	0.85 *	0.85 *	0.87 *
Ch*ab*			0.82 *	0.84 *	0.93 **	−0.85 *	−0.022	0.86 *	0.88 *	0.86 *	0.86 *
B				0.97 **	0.98 **	−0.81 *	−0.124	0.85 *	0.82 *	0.98 **	0.98 **
Bn					0.97 **	−0.87 **	−0.135	0.83 *	0.81 *	0.85 *	0.92 **
Bl						−0.94 **	−0.118	0.95 **	0.86 *	0.97 **	0.95 **
βC							0.77 *	0.96 **	0.94 **	0.92 **	0.97 **
AsA								0.86 *	0.95 **	0.97 **	0.82 *
TP									0.95 **	0.98 **	0.98 **
TF										0.87 *	0.99 **
AP (DPPH)											0.96 **

Ch*b*, chlorophyll *b;* Ch*a*, chlorophyll *a;* B, betacyanins; Ch*ab,* chlorophyll *ab;* Bl, betalains; Bn, betaxanthins; βC, β-carotene; AsA, ascorbic acid; TF, total flavonoids; AP, antiradical potential; TP, total polyphenols; *, ** significant at the 5% and 1% levels, respectively.

## Data Availability

Data is contained within the article.
